# Social Participation Among Older Immigrants: A Cross-Sectional Study in Nine Cities in Canada

**DOI:** 10.3390/healthcare13131478

**Published:** 2025-06-20

**Authors:** Sepali Guruge, Souraya Sidani, Jill Hanley, The ICOI Team

**Affiliations:** 1 Daphne Cockwell School of Nursing, Toronto Metropolitan University, Toronto, ON M5B 2K3, Canada; sidanis@yorku.ca; 2 School of Nursing, York University, Toronto, ON M3J 1P3, Canada; 3 School of Social Work, McGill University, Montreal, QC H3A 1B9, Canada; jill.hanley@mcgill.ca

**Keywords:** older adults, immigrant, social participation, solitary activities, community-based activities

## Abstract

**Background/Objectives:** Social participation is important for healthy aging but challenging for older immigrants because of factors such as the loss of cultural community, language and transportation barriers, ageism, and racism. This study aimed to examine (1) the type of social activities in which older immigrants from Arabic (Arab), Mandarin (East Asian), and Punjabi-speaking (South Asian) communities in Canada engage; (2) their desire for more participation in social activities; and (3) factors they perceive as preventing their engagement in more social activities. **Methods:** Using a cross-sectional design, we collected data, using existing measures, from 476 older immigrants between fall 2022 and winter 2023. Descriptive statistics were used to analyze the data. **Results:** More than 75% of participants reported engagement in three solitary activities (having a hobby, going on a day trip; and using the internet and/or email) and more than 85% participated in community-based activities with family inside and outside and with friends outside the household. Most (71%) expressed a desire to participate in more social activities in the community, but they were prevented from doing so due to factors such as language barriers or not wanting to go alone. **Conclusions:** Interventions are needed to facilitate community-based participation among older immigrants and improve their quality of life.

## 1. Introduction

Social participation or engagement is foundational for healthy or successful aging and a strategy for enhancing ‘aging in place’ [1,2]. It refers to older (≥60 years) adults’ involvement in activities that provide opportunities to interact, to various extents, with others in the community and/or society at large [3,4]. Such activities are diverse, ranging from community-based activities that directly promote active interactions with others (e.g., group physical activity, family events) to solitary yet socially oriented activities that provide opportunities to stay connected with others, community, and/or society (e.g., reading newspapers, voting). Older adults often engage in multiple activities, tailored to their personal preferences and needs [5]. Older immigrants, however, face particular barriers to engaging in social participation in their adopted country, such as the loss of access to their cultural community, language barriers, transportation barriers, ageism, and racism [6,7,8]. This topic has received limited research attention in Canada [9,10,11,12].

The objectives of this study are: (1) to describe the type of (solitary and community-based) social activities in which older immigrants from Arabic (Arab), Mandarin (East Asian), and Punjabi-speaking (South Asian) communities in Canada engage; (2) to examine their desire for more participation in social activities; and (3) to identify factors they perceive as preventing their engagement in more social activities. We begin the article with a review of the literature on older immigrants’ social participation, followed by a description of the study method. The results section provides a demographic description of the study sample before delving into the types of social participation in which older immigrants engage, the frequency of their participation, and the barriers they reported. Discussion explores the relative importance of social activities undertaken alone, and with family, friends, and the broader community, making the argument that it is important to take all forms of social activities into account alongside barriers, if we hope to understand older immigrants’ social participation. We conclude with a discussion of how service providers might apply this understanding in the design and delivery of interventions that can successfully support the level of social participation desired by older immigrants.

This article makes an original contribution to the literature on the social participation of older immigrants by including their engagement with family, friends, religious and co-ethnic groups, as well as the wider society. Notably, our study is the first in recent years to consider specific ethnic groups of older immigrants in research in particular cities (e.g., it is the first study on Mandarin-speaking immigrant older adults in Quebec since the 1980s).

## 2. Literature Review

In Canada, older immigrants represent 30% of the older adult population [13]. Recent years have witnessed an increased influx of immigrants from Arab, East Asian, and South Asian countries, and they are also the three largest immigrant communities in Canada [14,15].

The benefits of social participation—whether solitary, with family, friends, or community—are well supported empirically, encompassing improvement in the physical and mental domains of health. Social participation has been found to maintain cognitive function and prevent disability [1,16]. It also improves the sense of self-development and self-fulfillment, psychological comfort, satisfaction with life, and quality of life, and it reduces the feeling of loneliness [4,17,18]. Yet older (60+ years) immigrants face challenges that limit social participation in the host country and, therefore, may not experience its health benefits. Although studies in the U.S. have investigated social participation among Chinese [8,16], Korean [5], and other older immigrants, no similar research has recently been undertaken in Canada [12].

Different factors, including language and cultural barriers, physical and mental health, personal and family responsibilities, safety concerns, and access to transportation are often cited as factors affecting older immigrants’ ability to engage in social activities such as: interacting with friends and neighbors, accessing social groups, and attending cultural gatherings and events [6,7,8,16]. Older immigrants from Arab, East Asian and South Asian communities in Canada, in general, tend to have low proficiency in the country’s official languages, which alongside of experience of discrimination and racism, may result in preference to socialize with members of their family and/or with others from the same/similar cultures, with the opportunity to share common interests, knowledge, and social customs [19]. Issues with access to transportation may limit their engagement in social and/or cultural activities [20] and increase their involvement in solitary social activities.

In sum, these barriers may force older immigrants to engage in socially-oriented solitary activities (e.g., reading newspapers to stay abreast of current affairs; using social media to interact with acquaintances, friends, and family) more so than family-, friends-, or community-based social activities. Notably, however, solitary activities may not involve active interactions with groups, and they can be limited in enhancing older immigrants’ social connectedness with the community or society. Community-based social activities, in contrast, are much more likely to promote group interaction and, therefore, social connectedness. Identifying the type of activities in which older immigrants engage, whether they desire more engagement, and their perception of factors that limit their participation in more activities is therefore useful in generating knowledge that would inform the development of interventions or strategies aimed at enhancing their social participation.

## 3. Research Design and Methods

### 3.1. Design

This study was part of the Inclusive Community for Older Immigrants (ICOI) project (for details, visit www.icoi.ca), which consists of a collaboration between academic and community agency partners. This article reports on the data from the initial phase of the ICOI project that focused on generating an understanding of older immigrants’ experiences of social isolation, with the ultimate goal of developing, implementing, and evaluating interventions for promoting social connectedness. Arabic, Mandarin, and Punjabi-speaking older immigrants were recruited in nine Canadian cities.

This study used a cross-sectional design to collect data on older immigrants’ social participation. Several strategies were incorporated to enhance older immigrants’ enrollment. These included: (1) hiring and training research staff who are bilingual and bicultural, to promote a sense of trust and facilitate communication with older immigrants [21]; (2) offering participants the choice of completing the questionnaire in their preferred language (English, French, Arabic, Mandarin, or Punjabi), and the method (self-completion, research staff administered) and format (in-person, via technology, electronic or hard copy) of administration; and (3) providing incentives in the form of $30 gift card and $10 reimbursement for transportation. The questionnaire was translated by bilingual research assistants, and the translated version was reviewed and approved by bilingual researchers or community leaders and service providers.

### 3.2. Setting and Sample

The sample included older immigrants residing in nine cities within four provinces in Canada. Within each province, small/medium-sized and large metropolitan cities were selected: Gatineau and Montréal in Québec; Hamilton, London, and Toronto in Ontario; Calgary and Edmonton in Alberta; and Victoria and Vancouver in British Columbia.

The participant eligibility criteria were: (1) 60 years of age or older; (2) ability to provide oral or written consent, assessed by items developed and validated by Resnick et al. [22] to determine older immigrants’ understanding of the study participation expectations; (3) born outside Canada; and (4) self-identification with one of the selected immigrant communities: Arabic-speaking (Arab), Mandarin-speaking (East Asian), or Punjabi-speaking (South Asian) communities.

Following research ethics board approvals (Toronto Metropolitan University REB #2021-545, dated 28/02/2022 and REBs in all other eight cities), participants were recruited using active and passive strategies reported as successful with immigrants [23,24]. The specific recruitment strategies were chosen in collaboration with the staff at partnering community agencies within each city. Common strategies involved the following: referral by service providers at health, social, and settlement service agencies; participants informing others about the study; posting flyers in places frequented by older immigrants, such as ethnic grocery stores; and putting advertisements on social media frequently used by members of the respective immigrant communities.

Consistent with quota sampling, the number of participants varied across cities, based on the sampling pool available for each immigrant community within each city; that is, 90 or more participants were included in large cities where all three immigrant communities were recruited, and approximately 30 to 60 participants were included, respectively, in small/medium-sized cities where one to two communities were selected. The total number of participants who enrolled and completed the questionnaire was 476. This sample size is adequate to generate robust estimates of rates of engaging in social activities [25].

### 3.3. Measurement

All variables of interest were measured with items used in previous research involving older immigrants. The content of the items was validated for relevance and comprehension by older immigrants, as reported by the respective authors. Where applicable, the wording of the items was revised for consistency with the Canadian context (e.g., taking holidays in Canada, provincial elections). The translated items were reviewed and validated by bilingual and bicultural academic and clinical researchers affiliated with the ICOI.

Socio-demographic characteristics (age, gender, education, marital status, work status, financial condition, living arrangement, ethnicity, proficiency in the host country languages, and length of residence) were assessed using items developed from pertinent literature.

Engagement in solitary social activities was assessed with seven items (Table 1) utilized by Park et al. [5] and ten items by Kate et al. [26]. These activities included: reading newspapers; having a hobby or pastime; taking holidays inside or outside Canada; taking a day trip; using the internet or email; and voting in local, provincial, or national elections. Participants indicated whether or not (yes/no) they engage in each activity.

Participation in community-based activities was measured with 10 items (Table 2), used in Tang et al. [8], Tomioka et al. [27], Dawson-Townsend [28], and Aroogh and Shahboulaghi [29]. The activities consisted of attending family events inside and outside the household; meeting with friends outside the household; attending religious services; engaging in sports, educational or cultural clubs, neighborhood or community or professional associations, volunteer work, and recreational activities, in groups. For each activity, participants reported the frequency of engagement, with response options ranging from *never*, *at least once a year*, *at least once a month*, *at least once a week*, to *at least once a day*.

The desire for participation in more activities and factors preventing participants from more engagement were measured by items developed for the study; the items’ content was derived from the literature and validated by the project’s steering committee. Participants were asked whether or not (yes/no) they felt like they wanted to participate in more social, recreational, or group activities, and whether or not (yes/no) each of the 12 factors (Table 3) prevented them from doing so.

### 3.4. Data Analysis

Preliminary analysis showed no significant association between geographic location and the variables of interest; therefore, the main analysis was conducted on data obtained from participants across all nine cities. For the main analysis, descriptive statistics were used to analyze the data. The frequency distribution and percentage are reported to indicate the activities in which participants engage, their desire for more participation, and the factors they perceive as preventing them from doing so. The mean and standard deviation were estimated for continuous socio-demographic characteristics such as age.

## 4. Results

### 4.1. Socio-Demographic Characteristics

On average, participants were 69.8 (±6.92) years old. More than half (59.2%) of the participants were women, and 40.9% were men. They varied in their educational qualifications: 9.8% completed primary school; 10.6% had some secondary school; 14.5% completed high school; 22.8% had a college diploma or trade certificate; 31.2% had a Bachelor’s degree, 9.0% had a Master’s degree, and 2.1% had a PhD. The majority of participants (77.6%) were married, and 22.4% were not (14.1% widowed, 5.3% divorced, 1.8% separated, and only 1.3% were never married/single).

Slightly more than three-quarters (79.6%) of participants were retired. They rated their financial condition ‘below average’ (49.9%), ‘average’ (43.5%), or ‘above average’ (6.6%). A large percentage (78.9%) of participants reported living with family members (spouse, children, and/or grandchildren).

Participants included more Mandarin-speaking (55.6%) than Arabic (27.1%) or Punjabi (17.3%) speaking older immigrants. They had been in Canada for a mean of 16.5 (±13.47) years; 69.0% of participants had a length of residence less than or equal to 20 years. In general, participants had limited proficiency in Canada’s official languages, with mean scores of 1.64 (±1.10; range = 0–4) for English and 0.31 (±0.83; range = 0–4) for French.

### 4.2. Social Participation

More than 75% of participants reported engagement in three solitary social activities; these were: having a hobby or a pastime, going on a day trip in the past 12 months, and using the internet and/or email. About 57% had taken a holiday in Canada in the past 12 months (Table 1).

Participants’ engagement in community-based activities varied. The overwhelming majority indicated their involvement in the following: family-based activities inside the household (94.5%); family-based activities outside the household (88.3%); and friendship-based activities outside the household (88.4%). Also, participants reported their engagement in sports or physical activity (60.2%); religious activities (62%); recreational activities (56.6%); educational and cultural activities (56.6%); neighborhood, community or professional association (49.9%); volunteer or charity work (42.3%); and service club or fraternal organization (18.0%) activities. However, the participants often reported engaging in these activities at a low frequency (monthly or yearly) (Table 2).

Most (71.2%) participants expressed a desire to participate in more social activities, particularly those with direct interactions and outside of their homes. Several factors prevented them from doing so (Table 3). The factors were, in descending order: not wanting to go alone (57.6%), location of activities being too far (56.3%), language barriers (55.1%), transportation issues (53.9%), activities not available in the area (53.0%), location of activities is not physically accessible (47.9%), cost issues (42.6%), time of activities not being suitable (42.5%), having personal or family responsibilities (37.6%), health conditions/limitations (34.0%), safety concerns (30.8%), and being too busy (23.9%).

## 5. Discussion

The results of this study indicate that a large majority of study participants engage in both solitary and community-based social activities. However, given the limited benefits for solitary social activities noted above, here we explore the relative importance of social activities undertaken alone, with family, with friends, and with the broader community, as reported by participants. Taking into account all forms of social participation and their corresponding potential barriers is necessary to develop a fulsome understanding of older immigrants’ experiences and desires related to social participation.

### 5.1. Solitary Activities

The most common solitary activities participants engaged in were having a hobby or pastime, going on a trip, and using the internet or email. Physical health, harsh winter, language barriers, and transportation barriers, in particular, force some immigrant older adults to remain at home and engage in solitary activities such as hobbies (e.g., reading a book or watching a movie). These solitary activities can be limited in helping overcome the feeling of loneliness or isolation. Literature also shows that advanced age, limited education, and poor health status can shape older adults’ engagement in social activities [5]. Similarly, engagement in other somewhat solitary activities, such as visiting museums, may depend on education level, language proficiency, social support, and income [30].

### 5.2. Family-Based Activities

Our study participants reported engaging in family-based activities, both within and outside the household, to a greater extent than other types of community activities. Family-based activities inside the household included small get-togethers, meals, and/or reunions. Family-based activities outside the household included small get-togethers, meals outside of the household, weddings, religious celebrations, and/or reunions. The significant involvement in family-based activities is likely related to the fact that a majority of participants were married and living with family at the time of the study. The focus on family-based activities may also be related to the fact that about 70% of our study participants had been in Canada for less than 20 years and reported limited English/French language proficiency. Further, our study participants belong to ethnocultural and religious communities that place a high value on collectivism and focus on family and family cohesion [8,17,31,32]. Given the average age of our study participants and their length of stay in Canada (suggesting that many arrived in their 50s or later), it is very likely that a majority of participants may have arrived for family reunification purposes or to support their adult children with grandchild care and/or household work. As such, they may have limited time for pursuing social activities of their own outside the household [33,34].

### 5.3. Involvement with Friends

A majority of participants reported engaging in activities with their friends. These activities included get-togethers, meals outside the household, and reunions. These friendships often involve those within the same language group, and as such, can help promote a shared appreciation of ethnocultural values and beliefs, a sense of belonging, safety, and security, especially for marginalized and/or racialized groups. Chen et al.’s [31] results show that relatively recently arrived older immigrants (e.g., from East Asia) and those who face language barriers identify friends and neighbors who speak the same language as their primary sources of social connection and support.

### 5.4. Community-Based Activities

Approximately half of our study participants were engaged in sports/physical, religious, recreational, educational, cultural, neighborhood, and professional activities. About 40% of our study participants reported engaging in volunteer and charity work. This level of community-based social participation may be explained by participants’ socio-demographic characteristics. For example, more than 50% of participants had a college diploma, trade certificate, and/or at least one degree, as well as reported having average or above average financial status, all of which are noted in the literature as potential facilitators of social engagement [7,16]. Among the top community-based activities identified by our study participants was engagement in religious activities. Religious/faith-based affiliations are known to create a sense of belonging in a foreign land [31,35], which can act as a protection against discrimination [18]. Participants’ involvement in recreational activities (e.g., choirs, bridge, cards, other games) and service club or fraternal organization activities (such as Lions Club, Rotary, Kiwanis Club, Royal Canadian Legion, or Foresters) was minimal. It is possible that these may not be culturally relevant to Arabic, Mandarin, and Punjabi-speaking older adults.

### 5.5. Frequency of Engagement

While participants reported engaging in a range of community-based activities, the frequency with which they engaged in such activities varied considerably. Participants reported engaging in religious activities as well as sports or physical activities on a daily or weekly basis more than all other types of activities, indicating that such settings can be important sites in which social connections might be further encouraged and expanded. Participants’ expressed desire to participate in more social activities, particularly those outside the home, may be connected to this low frequency of engagement in other types of community-based activities.

### 5.6. Factors Preventing Social Participation

Participants identified a range of factors that prevented them from being more socially engaged. These were as follows: not wanting to go alone, location, language barriers, transportation issues, cost issues, time of activities, personal/family responsibilities, health conditions/limitations, safety concerns, and being too busy. The most common barriers are related to the activities’ location, language, and transportation. Language and transportation are common barriers noted in the literature as preventing immigrant older adults from engaging in community-based social activities [6,7,8]. Location is a key factor that can promote or prevent access to community-based activities. The current housing crises in most cities are pushing more and more immigrants (including older immigrant members of households) to suburban areas or the outer boundaries of the cities, which often do not have ready access to public transportation systems and walkable neighborhoods, which in turn prevent older immigrants from venturing out on their own to engage in community-based activities [34]. For example, attending activities at an ethnic senior center, where language barriers would be overcome, may not be possible in such areas with limited ethnic concentration. Further, economic insecurity and disadvantaged neighborhoods can hinder older immigrants’ social participation [16,36].

### 5.7. Study Limitations

Despite extensive recruitment, the sample may represent older immigrants who are more actively involved in social activities and thus are connected with others. Social desirability could have influenced participants’ responses. It is also possible that the measures, even though adapted from relevant literature, excluded other types of social activities and influential factors. Research, using qualitative methods, is needed to further explore older immigrants’ experiences, barriers, and facilitators of social participation.

## 6. Conclusions

Older immigrants comprise a significant percentage of older adults in Canada who face many barriers to social participation, and as a result, experience social isolation and loneliness. Understanding the types of social activities they engage in as well as the factors that prevent their social participation is important. Social participation must be understood as being on a continuum that ranges from solitary activities to family- and community-based activities. Our study findings demonstrate that families play a critical role in ensuring immigrant older adults’ social participation. However, placing the onus on family members can limit older immigrants’ engagement in community-based activities, which in turn limits older immigrants from building broader and more diverse social connections. Individuals tend to engage in multiple and diverse social activities based on their diverse socially constructed identities, personal and family preferences, ethnocultural values and beliefs, neighborhood factors, as well as structural barriers that prevent or limit their social engagement. Primary reliance on family for social connection and engagement is not only cultural but also a response to structural barriers older immigrants face. Community-based social participation may be facilitated by co-ethnolinguistic community organizations, which can help eliminate language and cultural barriers to social participation while fostering a sense of belonging. Coaching of immigrant older adults in the use of public transportation would be an important support for those feeling intimidated by the system. Formal health, social, and settlement service agencies can facilitate cross-cultural community-based activities that integrate immigrant and non-immigrant communities—in particular by offering linguistic supports—to promote social inclusion, cohesion, and integration.

## Figures and Tables

**Table 1:** Engagement in solitary social activities.

Item	No		Yes	
	N	%	N	%
I read a daily newspaper	350	54.9	287	45.0
I have a hobby or pastime	115	17.9	525	82.0
I have taken a holiday in Canada in the last 12 months	271	42.6	365	57.3
I have taken a holiday outside Canada in the last 12 months	419	66.1	214	33.8
I have gone on a daytrip or outing in the last 12 months	155	24.4	480	75.5
I use the internet and/or e-mail	128	20.0	511	79.9
I voted in the last federal, provincial, or municipal election	390	62.1	238	37.9

**Table 2:** Engagement in community-based activities.

Item	Never %	At Least Once a Year %	At Least Once a Month %	At Least Once a Week %	At Least Once a Day %
Family-based activities inside the household	6.5	16.7	27.7	28.3	20.5
Family-based activities outside the household	11.6	35.6	35.3	15.0	2.3
Friendship-based activities outside the household	11.5	31.7	37.2	16.5	3.0
Religious activities	38.0	16.7	12.8	26.5	5.8
Sports/physical activities for leisure	33.7	15.4	17.0	22.7	11.0
Educational and cultural activities	43.4	24.5	16.8	12.0	3.1
Service club or fraternal organization activities	81.9	8.7	5.8	2.6	0.8
Neighborhood, community or professional association social events	50.0	22.3	15.6	10.5	1.4
Volunteer or charity work	57.6	21.7	11.9	6.7	1.9
Other recreational activities with other people	43.3	16.4	19.16	15.5	5.5

**Table 3:** Factors preventing participation in more activities.

Factor	No		Yes	
	N	%	N	%
Don’t want to go alone	240	42.3	327	57.6
Location is too far	244	43.6	315	56.3
Language related reasons	264	44.9	324	55.1
Transportation problems	275	46.0	322	53.9
Activities not available in the area	266	46.9	301	53.0
Location not physically accessible	289	52.0	266	47.9
Cost	329	57.3	245	42.6
Time of the activities not suitable	318	57.5	235	42.5
Personal or family responsibilities	349	62.3	211	37.6
Health condition/limitations	381	65.9	197	34.0
Afraid or concerns about safety	388	69.1	173	30.8
Too busy	426	76.0	134	23.9

## Data Availability

Consent for public use of data was not obtained from the study participants, so it cannot be openly shared.
